# Disseminated Histoplasmosis in a Patient With HIV Infection Untreated for 23 Years

**DOI:** 10.7759/cureus.101167

**Published:** 2026-01-09

**Authors:** Andrea N Milton, Tobe Momah

**Affiliations:** 1 Family Medicine, University of Mississippi Medical Center, Jackson, USA

**Keywords:** aids complications, antiretroviral nonadherence, caseating granulomas, disseminated histoplasmosis, hiv advanced disease, immunosuppression, opportunistic fungal infection, retroperitoneal lymphadenopathy, severe cd4 depletion, stigma and hiv care

## Abstract

Histoplasmosis is a potentially life-threatening opportunistic fungal infection in patients with advanced human immunodeficiency virus (HIV), particularly in endemic regions such as the Ohio and Mississippi River valleys. Despite advances in antiretroviral therapy, delayed diagnosis, lack of linkage to care, and treatment nonadherence continue to contribute to severe opportunistic infections in individuals with untreated HIV.

We present the case of a 43-year-old woman with a 23-year-long history of untreated HIV infection who presented with fever, weight loss, diarrhea, and malaise. Laboratory evaluation revealed a viral load of 327,000 copies/mL and severe immunosuppression with a Cluster of Differentiation (CD4) count of 8 cells/µL, hyponatremia, and elevated liver transaminases. Computed tomography imaging demonstrated extensive mesenteric and retroperitoneal lymphadenopathy. Biopsy of a retroperitoneal lymph node revealed granulomas containing fungal organisms consistent with disseminated histoplasmosis. The patient was initially treated with empiric broad-spectrum antibiotics and intravenous amphotericin B, followed by long-term itraconazole therapy and initiation of antiretroviral treatment, resulting in clinical and immunologic improvement.

This case highlights the direct relationship between prolonged untreated HIV infection, advanced immunosuppression, and disseminated histoplasmosis, while underscoring the impact of stigma and barriers to care on delayed diagnosis and disease severity. Early HIV diagnosis, timely linkage to care, and sustained patient engagement remain essential to preventing severe opportunistic infections.

## Introduction

Histoplasmosis is a fungal infection caused by *Histoplasma capsulatum* and remains a significant opportunistic infection among individuals with advanced human immunodeficiency virus (HIV) and acquired immunodeficiency syndrome (AIDS), particularly in endemic regions such as the Ohio and Mississippi River valleys [1,2]. The risk of disseminated histoplasmosis increases substantially in patients with severe immunosuppression, most commonly when CD4 counts fall below 150 cells/µL, and especially below 50 cells/µL [1,3]. In immunocompromised hosts, disseminated disease may involve the gastrointestinal tract, lymphatic system, liver, spleen, and bone marrow, and is associated with significant morbidity and mortality if not promptly recognized and treated [3,4].

Despite advances in antiretroviral therapy, delayed diagnosis, lack of linkage to care, and treatment nonadherence continue to contribute to severe opportunistic infections in patients with untreated HIV [4,5]. This case report describes disseminated histoplasmosis in a patient with more than two decades of untreated HIV infection and highlights the clinical consequences of prolonged immunosuppression, barriers to care, and the importance of early diagnosis and sustained engagement in HIV treatment.

## Case presentation

A 43-year-old African American female patient with a past medical history of HIV (diagnosed in 2002 during her first and only pregnancy), cervical dysplasia, status post (s/p) colposcopy, and recurrent sinusitis s/p adenoidectomy (2021), was seen in the outpatient setting for acute complaints of chills, malaise, body aches, decreased appetite, weight loss, nausea, fever, and diarrhea for the past three weeks. Patient had a workup done in the clinic, which revealed an HIV viral load of 327,000 viral copies (vc)/ml and a CD4 count of 8 cells/cmm. She reported being told she had HIV in 2002, when she was pregnant with her only child, but never received any treatment nor followed up with infectious disease specialists because of her fear of stigmatization and possible harm of the medicine to her then yet to be born child.

**Figure 1:**
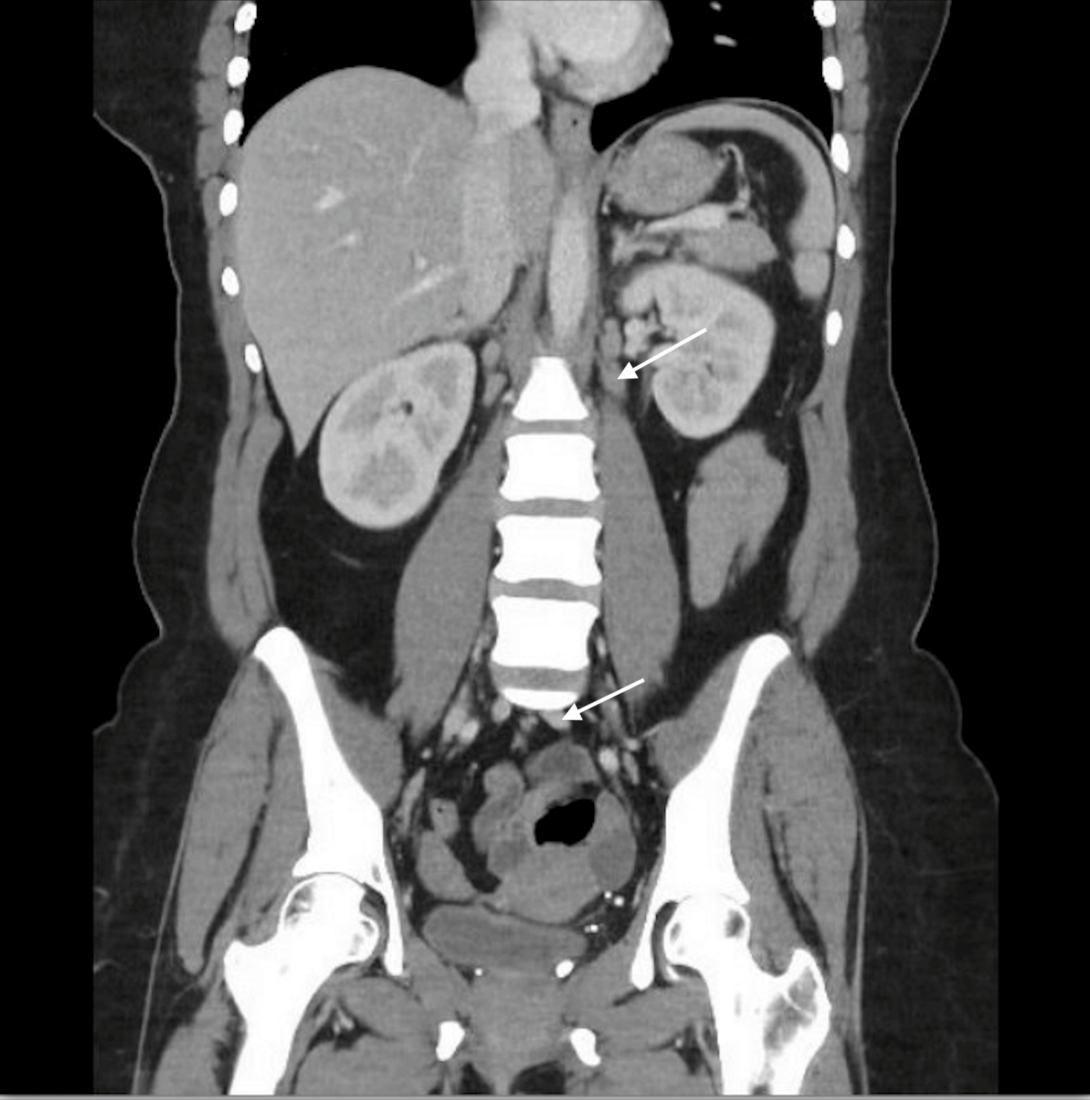
Coronal computed tomography image of the abdomen CT = computed tomography. Coronal CT image of the abdomen demonstrating enlarged retroperitoneal (upper arrow) and mesenteric (lower arrow) lymph nodes, consistent with disseminated histoplasmosis.

**Figure 2:**
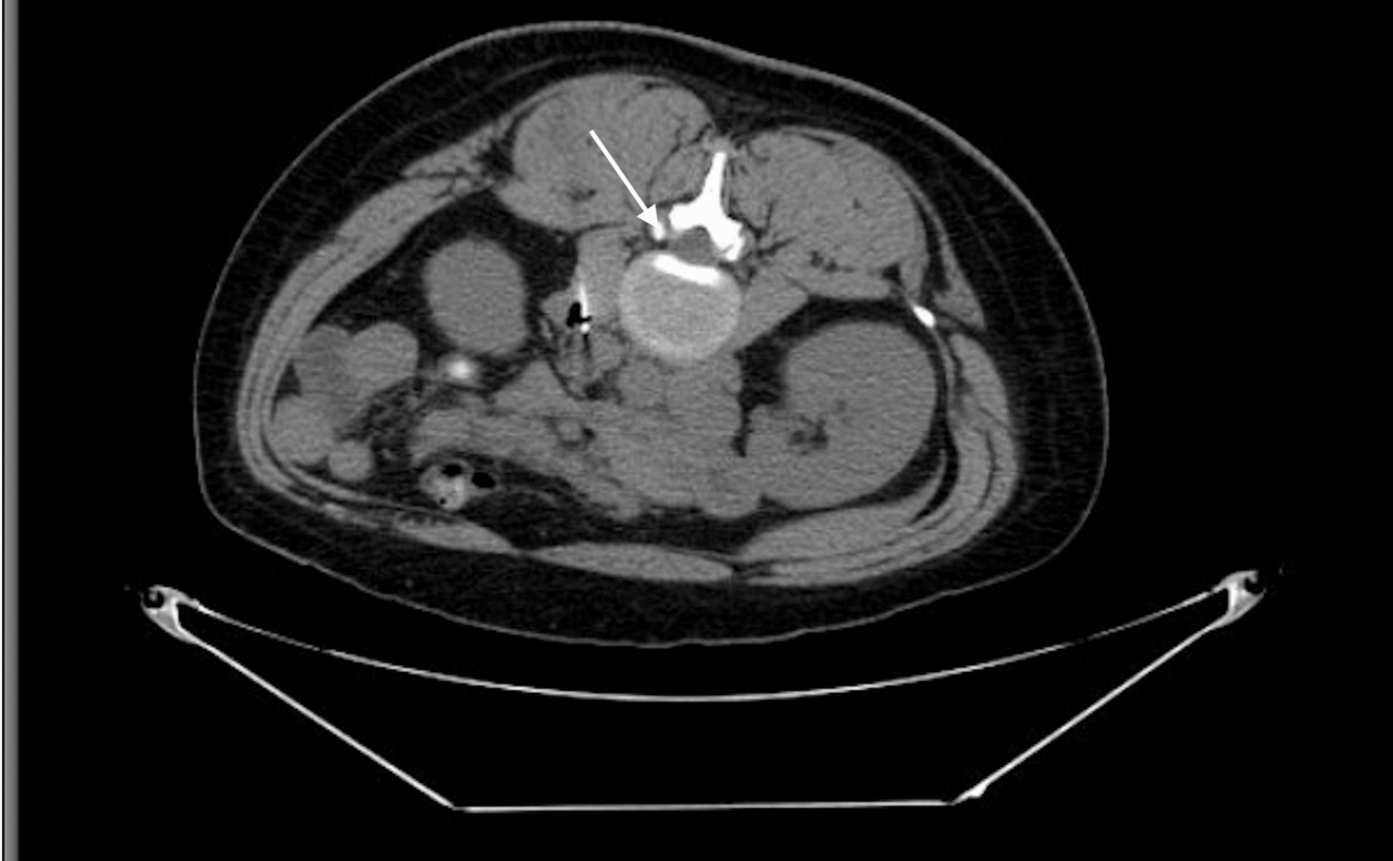
Axial computed tomography image of the abdomen CT = computed tomography. Axial imaging demonstrates a discrete left para-aortic retroperitoneal lymph node (arrow) measuring approximately 2.1 cm, corresponding to findings described in the case presentation.

She was admitted to a community hospital and started on bictegravir-emtricitabine-tenofovir alafenamide (Biktarvy) 50-200-25 mg tablet and sulfamethoxazole-trimethoprim (Bacrim DS) 400-80 mg, both once daily. Computed tomography of the abdomen demonstrated enteritis and pathologically enlarged mesenteric and retroperitoneal lymphadenopathy, including a discrete 2.1-cm left para-aortic retroperitoneal lymph node (Figures 1, 2).

Her vital signs were significant for fever (101.9°F) and tachycardia (105 beats/min). Laboratory evaluation was notable for hyponatremia (125 mmol/L; reference range 135-145 mmol/L), enteropathogenic *Escherichia coli* identified on stool culture, and elevated liver function tests with aspartate aminotransferase (AST) of 224 IU/L (reference range 6-34 IU/L) and alanine aminotransferase (ALT) of 94 IU/L (reference range 4-36 IU/L). The patient was started on intravenous antibiotics (piperacillin/tazobactam and vancomycin) and subsequently underwent CT-guided biopsy of a retroperitoneal lymph node.

Histopathology results from the retroperitoneal lymph node biopsy were positive for caseating granuloma with extensive histiocytic inflammation containing intracellular fungal organisms most consistent with Histoplasma. The results were negative for malignancy, and the patient was started on intravenous amphotericin B infusion 300 mg once daily for 14 days, and then continued afterwards on oral Itraconazole once daily for 12 months. Upon discharge, the patient has been compliant with her medications (including Biktarvy, Bactrim DS, and itraconazole) and has seen her HIV viral load reduced to 50 vc/ml and her CD4 count increased to 166 cells/cmm. She has also returned to work at a nearby factory (as a forklift operator), and denies any active sexual relationships.

## Discussion

Disseminated histoplasmosis is very common in Acquired Immunodeficiency Syndrome (AIDS) patients [1], with some studies noting a prevalence of 5% among AIDS patients in the Ohio/Mississippi valley and other reports noting a global incidence of approximately 100,000 cases in 2017 [2]. It is caused by the *Histoplasma capsulatum* fungus and in the United States of America (USA) is most commonly seen among those living in the surroundings of the Ohio and Mississippi river valleys [3].

There are two types of histoplasmosis in HIV/AIDS patients. These include acute cases which present with fever, body aches, malaise, headaches, chest pain, or cough alongside pulmonary, dermatological, or rheumatological findings [3]. The other type is disseminated histoplasmosis, which presents in elderly and immunosuppressed patients with more widespread systemic involvement (including bone marrow, liver, lymph nodes, spleen, and Gastrointestinal tract) [4].

Our patient had gastrointestinal and reticuloendothelial system involvement, and as a result, we diagnosed her with disseminated histoplasmosis. She required aggressive treatment using intravenous amphotericin B and is now on oral itraconazole for the following 12 months. Her lack of HIV treatment, post-diagnosis in 2002, most likely resulted in her disseminated histoplasmosis condition, and her case highlights the need to connect every patient diagnosed with HIV to linkage of care and adequate follow-up [5]. She stated during her clinical visits that the stigma of entering an office dedicated to managing HIV/AIDS, and the fact that one of the principal staff knew her brother dissuaded her from attending to her health at that particular office. 

The need to destigmatize HIV treatment in our medical system cannot be overemphasized. Our patient was infected by her child’s partner, and their inability to connect to care inadvertently resulted in the increased costs of care (as seen from our index patient’s admission costs), and complications such as disseminated histoplasmosis arising in our patient.

## Conclusions

This case illustrates disseminated histoplasmosis as a severe opportunistic infection arising in the setting of prolonged, untreated HIV infection and advanced immunosuppression. At presentation, the patient’s profoundly low CD4 count (8 cells/µL) reflected longstanding immune dysfunction, predisposing her to disseminated disease. Cross-sectional imaging demonstrated extensive mesenteric and retroperitoneal lymphadenopathy, including a discrete para-aortic lymph node, and tissue biopsy confirmed granulomatous inflammation with fungal organisms consistent with *Histoplasma capsulatum*. Together, these findings directly link prolonged untreated HIV infection, severe CD4 depletion, and the development of disseminated histoplasmosis.

Initiation of antifungal therapy with intravenous amphotericin B followed by long-term itraconazole, along with antiretroviral therapy, resulted in clinical improvement, virologic suppression, and immunologic recovery, as evidenced by rising CD4 counts. This clinical course underscores the causal relationship between immune restoration and disease resolution. Additionally, this case highlights the role of delayed linkage to care and perceived stigma as contributors to prolonged untreated HIV infection and advanced disease at presentation.

Clinicians practicing in histoplasmosis-endemic regions should maintain a high index of suspicion for disseminated histoplasmosis in patients with advanced or untreated HIV who present with systemic or gastrointestinal symptoms. Early recognition, prompt imaging, tissue diagnosis when lymphadenopathy is present, and timely initiation of antifungal and antiretroviral therapy are essential to reduce morbidity and mortality. Integrating HIV services into broader, less stigmatizing clinical environments may further improve patient engagement, continuity of care, and long-term outcomes.
